# Evaluation of routine health monitoring for metabolic disorders in patients with serious mental illness on psychotropic medications: a study from Ethiopia

**DOI:** 10.1186/s12888-024-06266-1

**Published:** 2024-11-12

**Authors:** Tirsit Ketsela Zeleke, Abel Hedato Teshome, Meron Tademe Assefa, Gashaw Sisay Chanie, Rahel Belete Abebe

**Affiliations:** 1 Department of Pharmacy, College of Health Sciences, Debre Markos University, Debre Markos, Ethiopia; 2 Department of General Surgery, College of Health Sciences, Debre Markos University, Debre Markos, Ethiopia; 3 Department of Laboratory Science, College of Medicine and Health Sciences, Haromaya University, Harer, Ethiopia; 4 Department of Clinical Pharmacy, School of Pharmacy, College of Medicine and Health Sciences, University of Gondar, Gondar, Ethiopia

**Keywords:** Mental illness, Psychotropic medication, Health monitoring, Ethiopia

## Abstract

**Background:**

Compared to the overall population, patients with mental health problems are more likely to experience concurrent physical illnesses, poorer health outcomes, and mortality. Psychotropic medications, which are the pillars in the management of mental health conditions, are associated with adverse effects such as weight gain, an increased level of glucose, and elevated circulating lipid levels, all of which contribute to metabolic disorders. Inadequate health monitoring may led to suboptimal interventions and worsening of these conditions. However, there is a lack of studies assessing routine health monitoring practices for metabolic disorders and their determinants among patients with serious mental illnesses taking psychotropic medications in Ethiopia. This study aimed to evaluate routine health monitoring for metabolic disorders and its determinants in patients with serious mental illnesses on psychotropic medications in Ethiopia.

**Method:**

A hospital based prospective follow-up study was conducted among patients with serious mental illness taking psychotropic medications who attended the outpatient psychiatry department at Debre Markos Comprehensive Specialized Hospital. Eligible participants were selected using a simple random sampling technique. Routine health monitoring was evaluated using guidelines and previous literature. Binary logistic regression was employed to identify the determinants of routine health monitoring, with statistical significance determined by a p-value of less than 0.05 and a 95% confidence interval (CI).

**Results:**

The overall routine health monitoring practice for metabolic disorders was found to be sub-optimal. Vital signs were the most commonly assessed parameters. Key determinants of routine health monitoring included participants aged 45 and above (AOR (95% CI): 2.82 (1.34–5.92), having social insurance (AOR (95% CI): 2.94 (1.86–4.64), availability of laboratory tests at the hospital (AOR (95% CI): 3.46 (2.16–5.55), and reporting of medication-related side effects (AOR (95% CI): 1.96 (1.21–3.17)).

**Conclusion:**

Routine health monitoring for metabolic disorders in patients with serious mental illnesses attending the outpatient psychiatry department was inadequate. Health care providers should give more attention to younger patients, those without health insurance, and who are not reported side effects. These findings provide crucial insights for improving routine health monitoring and promoting better health outcomes.

**Supplementary Information:**

The online version contains supplementary material available at 10.1186/s12888-024-06266-1.

## Introduction

Serious mental illnesses (SMI) refer to a group of chronic mental disorders, including schizophrenia, bipolar disorder, and major depressive disorder with psychotic features [1]. SMIs can also encompass anxiety disorders, eating disorders, and personality disorders when the level of functional impairment is severe [2]. These conditions are characterized by a significant and ongoing impacts on individual’s cognitive, emotional, and social functioning, leading to a corresponding impairment in daily activities [3, 4]. SMI greatly affect an individual’s ability to perform routine tasks, often requiring continuous medical and psychological intervention [5, 6]. Globally, SMI contribute substantially to the burden of years lived with disability [7].

Health monitoring involves the regular assessment of various health parameters to guide the treatment of persistent or recurrent conditions. This process can be conducted by patients, health care providers, or a combination of both [8]. Compared to the general population, individuals with SMIs are more likely to experience concurrent physical illnesses, poorer health outcomes, and increased mortality [9–11]. Cardiovascular risk factors and metabolic disorders are prevalent among patients with SMIs, indicating the critical need for effective health monitoring [12–14].

The lifestyles of individuals with SMIs often contribute to these health risks, as they are frequently associated with poor dietary habits, obesity, high smoking rates, and illicit substance use [15, 16]. Additionally, psychotropic medications, which are essential for managing mental health conditions, are linked to adverse effects such as weight gain, elevated glucose levels, and increased lipid levels, all of which can lead to cardiovascular disease [17–20] and metabolic disorders [21]. Numerous antipsychotic medications increase the risk of obesity, dyslipidemia, type two diabetes, and other metabolic complications [22, 23].

Globally, hospital readmission rates are highest among individuals with chronic diseases, underlining the need for effective monitoring to reduce unplanned readmissions [24–26]. Routine visits for managing chronic diseases, including SMIs, typically involve monitoring to asses disease progression or complications [27]. Such assessments require careful consideration of what to monitor, when to conduct assessments, and how to adjust treatment strategies. Poor decisions in monitoring can lead to inadequate disease control, inefficient use of time, and potentially harmful treatment adjustments that pose risks to patient safety [28].

To slow disease progression, hospitals must implement a systematic approach to monitoring patient conditions and initiating necessary interventions [29]. Therefore, routine health monitoring is essential in hospital settings for the effective treatment of SMIs. Such practices can enhance the efficient use of medical staff and resources while improving overall patient care standard [30, 31].

Clinical encounters between patients and health care providers may include the monitoring of vital signs, physical examinations, and laboratory tests, which are integral components of health monitoring [32]. Guidelines recommend that patients experiencing metabolic disorders due to psychotropic medications should be transitioned to alternatives with lower risk of adverse effects on metabolic function [33, 34]. For instance, patients experiencing considerable weight gain or hyperglycemia are advised to switch from antipsychotic medications to alternatives with less detrimental metabolic impacts [35, 36]. However, without proper monitoring data, such interventions cannot be effectively implemented.

Despite the critical importance of routine health monitoring for metabolic diseases, that there is a lack of studies assessing these practices and their determinants among patients with SMIs in Ethiopia. The aim of this study is to evaluate routine health monitoring practices for metabolic diseases and their determinants among patients with SMIs in Ethiopia. The findings will be used to recommend health care providers to adopt evidence-based health monitoring practices and adhere to established guidelines.

## Methods and materials

### Study setting and design

A hospital based prospective follow-up study was conducted from June 1, 2022, to May 30, 2023 at Debre Markos comprehensive specialized hospital (DMCSH), Ethiopia.

### Source and study population

All adult patients with SMI who visited outpatient department of psychiatry at DMCSH were the source population, while those patients who visited psychiatry outpatient department during the data collection period were study population.

### Eligibility criteria

Patients with SMI who were newly diagnosed, those with follow-ups, and those taking at least one psychotropic medications were included in the study. Patients who were unable to communicate or had incomplete clinical documentation were excluded.

### Sample size determination

A single population proportion formula was used to estimate the sample size: considering the assumptions of a 95% confidence interval, 5% margin of error, and a 50% prevalence

$${\rm{n}} = {{\rm{z}}^2}{\rm{p }}(1 - {\rm{p}})$$

$$\mathrm{d}^2$$

$${\rm{n}} = {(1.96)^2}(0.5)\;(0.5) = 384$$

$${(0.05)^2}$$

Lastly, we add a 10% non-response rate which made our final sample size to be **422.**

### Sampling technique

We employed a proportional allocation method to select participants from the two primary diagnostic groups: schizophrenia and bipolar disorder. The proportional allocation was guided by previous hospital reports indicating the prevalence of these diagnoses within similar patient populations. These reports revealed that approximately 64.5% of patients diagnosed with serious mental illness had schizophrenia, while about 35.5% were diagnosed with bipolar disorder. To determine the number of participants in each diagnostic category, we applied the proportions derived from earlier reports to the final sample size. Specifically, 0.645 multiplied by 391 yielded approximately 252 schizophrenia patients, and 0.355 multiplied by 391 yielded approximately 139 bipolar patients. A simple random sampling procedure was then used to select participants within each diagnostic group.

### Study variables

The dependent variable was routine health monitoring for metabolic disorder. Independent variables.

Included sociodemographic parameters (sex, age, residence, educational level, monthly income, health insurance), medication related parameters (name, dose, and frequency of medication use), and clinical factors (diagnosis, presence of comorbid conditions, type of the comorbid disease, availability of laboratory tests at the hospital), and health care provider specialty.

### Operational definition

**Routine health monitoring for metabolic disorders**: refers to health monitoring practices that adhere to clinical guidelines and published literature recommendations [37–39] (See supplementary file 1).

### Data collection tools and procedure

A structured questionnaire was developed based on a review of literatures (See supplementary file 2), and was used by data collectors to extract relevant data. Four nurses and one supervisor were participated in the data collection process. The questionnaire was divided into four sections: sociodemographic parameters, medication-related parameters, clinical-related parameters, and health monitoring for metabolic disorders parameters. Clinical findings and prescribed medications were retrieved from medical records, while health monitoring practices were obtained through observation and medical records. Sociodemographic information was collected through interviews. Newly diagnosed patients with SMI who met the eligibility criteria were assessed. Health monitoring practices at the initiation of therapy and during follow-up visits were documented. Monitoring parameters such as weight and body mass index were not usually recorded; in this case, the findings were obtained through observations. Other parameters, including blood pressure, glucose level, lipid profile, complete blood count, thyroid function tests, and organ function tests, were taken from patients’ medical charts during follow-up visits.

### Routine health monitoring evaluation

Routine health monitoring was evaluated based on guidelines and previous literature [37–39]. Medication specific monitoring parameters were evaluated using additional recommendations from previous studies [39–42] (See supplementary file 1).

### Data quality assurance

The questionnaire was initially created in English and translated into Amharic. It was then back-translated to English to ensure consistency. Modifications were made based on the translation. Supervisors and data collectors were trained on the study’s objectives, methodology, and ethical considerations. A pretest was conducted at the comprehensive and specialized hospital of the University of Gondar among 21 patients with severe mental illness (5% of the overall sample size) to assess the clarity and sociocultural appropriateness of the questionnaire. Additionally, the investigator reviewed the data for completeness and consistency, while the supervisors provided on-site supervision throughout the data collection period.

### Data processing and analysis

Data were coded, checked for accuracy, and imported into Epi-data 4.6, then exported to SPSS version 26 for analysis. Frequencies and percentages were calculated for categorical variables (such as sex, residence, education, presence of comorbidities, medication parameters, and health care provider specialty), while means and standard deviations were used for continuous variables (such as age and monthly income). Binary logistic regression was employed to analyze the determinates of routine health monitoring. The Hosmer and Lemeshow goodness of fit test was used to assess the model’s fitness, and multicollinearity among independent variables was evaluated using the variance inflation factor, which was within an acceptable level of one to five. Variables with a p-value less than 0.2 in the bivariable analysis were entered into a multiple binary logistic regression model. Adjusted odds ratios (AORs) were used to report statistical associations, and a p-value < 0.05 was considered statistically significant. The results were presented using text, figures, and tables to summarize the findings.

## Result

### Sociodemographic characteristics of the study participants

A total of 391 participants were enrolled in this study, with a response rate of 92.6%. The mean age of the participants was 34 years with a standard deviation of ± 12.3. More than half of the participants (54.0%) were male, and 50.9% were from urban areas. Regarding education, 26.1% of the participants had a higher education level, while the average monthly income was 2,556 Ethiopian Birr (ETB), with a standard deviation of ± 1,253 ETB. The majority of the participants (61.9%) did not have health insurance (Table 1).

**Table 1:** Sociodemographic characteristics of patients with serious mental illness taking psychotropic medications at outpatient psychiatric department at DMCSH, Amhara, Ethiopia

Variable	Frequency (*n*)	Percent (%)
Age		
18–24	76	19.4
25–34	158	40.4
35–44	84	21.5
≥ 45	73	18.7
Sex		
Male	205	52.4
Female	186	47.6
Residence		
Rural	188	48.1
Urban	203	51.9
Educational status		
Illiterate	95	24.3
Literate with no formal education	85	21.7
Primary	65	16.6
Secondary	42	10.7
Higher	104	26.6
Monthly income in Ethiopian birr		
< 2556 ETB	257	65.7
≥ 2556 ETB	134	34.3
Health insurance		
No	242	61.9
Yes	149	38.1

ETB Ethiopian Birr

### Clinical parameters of the study participants

The majority of the participants (64.5%) had a diagnosis of schizophrenia. Most of the patients (70.6%) did not have any comorbid diseases. Among those with comorbid conditions, hypertension was the most common (40.0%), followed by human immunodeficiency virus (HIV) and diabetes mellitus in 26.9% and 23.5% of the patients, respectively. Additionally, more than half of the participants (59.8%) had access to laboratory tests at the hospital, while 52.7% received medications from a psychiatric nurse order (Table 2).

**Table 2:** Clinical parameters of patients with serious mental illness taking psychotropic medications at outpatient psychiatric department at DMCSH, Amhara, Ethiopia

	Variable	Frequency (*n*)	Percent (%)
Diagnosis			
	Schizophrenia	252	64.5
	Bipolar disorders	139	35.5
Presence of comorbid conditions			
	No	276	70.6
	Yes	115	29.4
Type of the comorbid disease			
	Hypertension	46	40.0
	HIV	31	26.9
	Diabetes mellitus	27	23.5
	Congested heart failure	14	12.2
	Others*	4	3.5
Availability of Laboratory tests at the hospital			
	No	157	40.2
	Yes	234	59.8
Health care provider specialty			
	Psychiatry nurse	206	52.7
	Psychiatrist	185	47.3

*Epilepsy, Asthma, Acute coronary syndrome HIV human immunodeficiency virus

### Medication and treatment parameters

A total of 534 medication prescriptions were issued to 391 patients at the outpatient psychiatry department. The most commonly prescribed group of medications were first generation antipsychotics (33.3%), followed by mood stabilizers (18.9%) and second-generation antipsychotics (17.4%). In terms of medication distribution, 41.7% of patients received first generation antipsychotics, while 22.5% and 14.8% received mood stabilizers and second-generation antipsychotics, respectively (Table 3).

**Table 3:** Frequency of medication prescriptions among patients with serious mental illness taking psychotropic medications at outpatient psychiatric department at DMCSH, Amhara, Ethiopia

	Frequency of medications per patient		Frequency of medications prescription	
Variables	Frequency (n)	Percent (%)	Frequency (n)	Percent (%)
Group of medications prescribed				
Antipsychotic				
First generation antipsychotics	163	41.7	178	33.3
Second generation antipsychotics	58	14.8	93	17.4
First generation + second generation	31	7.9	62	11.6
Mood stabilizer	88	22.5	101	18.9
Antipsychotic + mood stabilizer	37	9.5	78	14.6
Antipsychotic + anti-depressant	14	3.6	22	4.2
Total	391	100	534	100

Regarding specific medication prescription haloperidol was the most frequently prescribed medication (21.9%), followed by chlorpromazine, carbamazepine, and risperidone that held 19.8%, 17.6%, and 12.2% of prescriptions respectively. More than half of patients (58.8%) were prescribed monotherapy, and the majority of the respondents (67.3%) did not report medication related side effects (Table 4).

**Table 4:** Frequency of specific medication prescription and medication related parameters among patients with serious mental illness taking psychotropic medications at outpatient psychiatric department at DMCSH, Amhara, Ethiopia

	Variables	Frequency (*n*)	Percent (%)
Type of medications prescribed	Haloperidol	117	21.9
	Chlorpromazine	106	19.8
	Carbamazepine	94	17.6
	Risperidone	65	12.2
	Lithium	38	7.2
	Olanzapine	37	6.9
	Clozapine	32	6.0
	Sodium valproate	31	5.8
	Fluoxetine	9	1.7
	Amitriptyline	5	0.9
	Total	534	100
Medication regimen	Mono therapy	230	58.8
	Poly therapy	161	41.2
Medication related side effect	No	263	67.3
	Yes	128	32.7

### Routine health monitoring among the study participants

Routine health monitoring for metabolic parameters was inadequate for most patients. Among patients taking antipsychotics, the most commonly assessed monitoring parameter was blood pressure (93.7%), followed by body mass index (30.0%) and glucose level testing (25.0%). For those on mood stabilizers, blood pressure was monitored in 95.4% of cases, while body mass index was measured in 30.6%, and complete blood count tests were performed in 18.2%. Details and comprehensive data about the prevalence of routine monitoring parameters are shown in Table 5.

**Table 5:** Prevalence of routine health monitoring parameters and routine health monitoring among patients with serious mental illness taking psychotropic medications at outpatient psychiatric department at DMCSH, Amhara, Ethiopia

Group of medication	Number of patients taking medication	Routine health monitoring parameters		Number of patients received routine health monitoring	
				Frequency (n)	Percent (%)
Antipsychotics	303 (77.5%)	Vital signs			
			Blood pressure	284	93.7
		Body mass index/weight		91	30.0
		Glucose level		76	25.0
		Lipid profile		70	23.1
Mood stabilizers	88 (22.5)	Blood pressure		84	95.4
		Body mass index		27	30.6
		Kidney function tests			
			Serum creatinine/BUN	18	12.4
			Creatine clearance	18	12.4
		Liver function tests			
			SGOPT/SGPT	6	6.8
			Bilirubin	3	3.4
		Thyroid function test		Not done	Not done
		Electrolyte tests			
			Calcium	8	9.0
		Complete blood count		16	18.2
		Coagulation tests		4	4.5

BUN blood urea nitrogen, SGOT Serum glutamic oxaloacetic transaminase, SGPT (Serum glutamate pyruvate transaminase

### Determinates of routine health monitoring among study participants

The findings from the binary logistic regression analysis showed that sociodemographic parameters such as sex, residence, education, monthly income, as well as clinical and medication-related parameters, were not significantly associated with routine health monitoring. However, participants whose age is 45 years and older, having health insurance, availability of laboratory tests, and experiencing medication-related side effects were significant determinates. Participants aged 45 and above were 2.82 times more likely to receive routine health monitoring (AOR (95% CI): 2.82 (1.34–5.92). Participants who had health insurance had a 2.94 times greater tendency to get routine health monitoring compared to those who had no health insurance (AOR (95% CI): 2.94 (1.86–4.64). The other significantly associated variable was the availability of laboratory tests. The probability of patients accessing routine health monitoring increased by 3.46 when the laboratory tests were available at the hospital (AOR (95% CI): 3.46 (2.16–5.55)). Moreover, patients who experienced medication-related side effects had a 1.96 times higher rate of receiving routine health monitoring (AOR (95% CI): 1.96 (1.21–3.17)) (Table 6).

**Table 6:** Determinants of routine health monitoring among patients with severe mental illness taking psychotropic medications at outpatient psychiatric department at DMCSH, Amhara, Ethiopia

	Variable	Routine health monitoring done		Bi variable analysis			Multi variable analysis		
		Yes	No	P	COR	CI	P	AOR	CI
Age									
	18–24	24	52		1			1	
	25–34	67	91	0.11	1.59	0.89–2.84	0.18	1.53	0.81–2.88
	35–44	36	48	0.14	1.62	0.85–3.10	0.35	1.39	0.68–2.86
	>=45	42	31	0.00	2.93	1.50–5.73	0.00*	2.82	1.34–5.92
Sex									
	Male	94	111	0.27	1.25	0.83–1.87			
	Female	75	111		1				
Residence									
	Rural	86	102	0.33	1.21	0.81–1.82			
	Urban	83	120		1				
Education									
	Illiterate	41	54		1				
	Literate with no formal education	38	47	0.83	1.06	0.59–1.92			
	Primary	30	35	0.70	1.12	0.59–2.12			
	Secondary	16	26	0.58	0.81	0.38–1.70			
	Higher	44	60	0.90	0.96	0.55–1.69			
Monthly income in Ethiopian birr									
	< 2556 ETB	104	153		1			1	
	≥ 2556 ETB	65	69	0.12	1.38	0.91–2.11	0.24	1.32	0.82–2.12
Health insurance									
	No	80	162		1			1	
	Yes	89	60	0.00	3.00	1.96–4.58	0.00*	2.94	1.86–4.64
Diagnosis									
	Schizophrenia	83	106	0.50	1.33	0.57–3.06			
	Bipolar	44	59	0.59	1.26	0.52–3.03			
	Major depressive disorder	32	40	0.50	1.36	0.54–3.37			
	Generalized anxiety disorder	10	17		1				
Presence of comorbid condition									
	No	111	165		1			1	
	Yes	58	57	0.06	1.51	0.97–2.34	0.07	1.56	0.96–2.55
Type of medication prescribed									
	Antipsychotic	85	109	0.73	0.78	0.19–3.20			
	Antidepressant	55	72	0.71	0.76	0.18–3.19			
	Mood stabilizer	19	24	0.76	0.79	0.17–3.58			
	Antipsychotic + mood stabilizer	6	13	0.37	0.46	0.08–2.50			
	Antipsychotic + anti-depressant	4	4		1				
Medication related side effect									
	No	100	163		1			1	
	Yes	69	59	0.00	1.90	1.24–2.92	0.00*	1.96	1.21–3.17
Medication regimen									
	Mono therapy	97	`133		1			1	
	Poly therapy	72	89	0.21	1.10	0.73–1.66	0.18	1.35	0.86–2.14
Health care provider specialty									
	Psychiatry nurse	94	112		1	0.54–1.21			
	Psychiatrist	75	110	0.31	0.81				
Availability of Laboratory tests at the hospital									
	Not available	40	117					1	
	Available	129	105	0.00	3.59	2.31–5.59	0.00*	3.46	2.16–5.55

*Statically significant, AOR adjusted odds ratio, COR crude odds ratio, CI confidence interval

## Discussion

This study is the first of its kind, to our knowledge, that assessed routine health monitoring practice and its determinates in patients with SMIs taking antipsychotics in Ethiopia. A significant proportion of patients in this study were receiving first generation antipsychotics, with haloperidol and chlorpromazine being the most common. The frequent use of these medications is likely due to their affordability and availability in resource limited settings like Ethiopia. This finding is consistent with previous studies conducted in Gondar, Ethiopia, where first generation antipsychotics were also most frequently used for similar reasons [43]. Despite their effectiveness in managing psychosis, these medications are associated with a higher risk of extrapyramidal side effects [44], indicating the importance of careful clinical monitoring. Among second-generation antipsychotics risperidone and olanzapine were the most commonly prescribed in this study, aligning with findings from studies conducted in Kenya and Ghana [45, 46]. The preference for risperidone may be due to several factors, including its efficacy, tolerability, and suitability for treating a wide range of psychotic disorders, especially schizophrenia and bipolar disorder. Research has shown that risperidone effectively manages both positive symptoms, such as hallucinations and delusions, and negative symptoms, including apathy and social withdrawal, commonly observed in schizophrenia [47, 48]. Risperidone is an atypical antipsychotic with a lower risk of extrapyramidal side effects compared to first-generation antipsychotics, which may contribute to its frequent prescription in psychiatric settings [49]. This reduced risk is crucial for improving patient adherence, as motor side effects are often associated with discontinuation. The use of risperidone may also be influenced by its availability in various formulations, including long-acting injectable forms. Long-acting formulations are especially beneficial for patients with adherence challenges, as they reduce the frequency of dosing and provide consistent medication levels in the body [50]. However, while risperidone presents a lower metabolic risk compared to some antipsychotics, continuous monitoring remains essential to promptly address any emerging side effects, thus optimizing therapeutic outcomes and minimizing complications [51]. On the other hand in this study olanzapine was frequently prescribed medication following risperidone. The possible reason might be due to its lower risk of extrapyramidal symptoms and its efficacy in treating both positive and negative symptoms of psychosis. However, second-generation antipsychotics, including olanzapine, are known to cause metabolic side effects such as weight gain, increased risk of diabetes, and dyslipidemia [52]. These metabolic risks show the critical need for routine health monitoring in patients receiving psychotropic medications.

The overall evaluation of routine health monitoring practices among patients with SMI taking psychotropic medications in this study revealed a significant inadequacy. Only a small proportion of participants received regular assessments of key metabolic parameters, which is concerning given the known risks associated with these medications. This finding is consistent with a study from northeast England, which reported inadequate routine monitoring of metabolic conditions in the majority of patients with SMI [10]. Patients diagnosed with SMIs, such as schizophrenia and bipolar disorder, are particularly vulnerable to developing metabolic problems [53]. The prevalence of metabolic syndrome is markedly higher in this population, driven by a combination of factors, including the side effects of antipsychotic medications, lifestyle choices, and the physiological impacts of their mental illnesses. For instance, between 36% and 50% of individuals severe depression meet the criteria for metabolic syndrome [54]. For this reason, various clinical guidelines and recommendations have emphasized the critical importance of routine metabolic monitoring for patients with SMI [35, 55]. These guidelines advocate for regular assessments of weight, blood pressure, glucose levels, and lipid profiles to identify and address potential metabolic issues early. Despite these recommendations, studies report that many patients with SMI have undiagnosed metabolic disorders, often due to minimal intervention and insufficient monitoring, leading to serious health complications over time [56]. Failure to detect and manage these issues contributes to poor health outcomes in this vulnerable population [10]. Therefore, enhancing routine health monitoring practices is essential to improve the overall quality of care and reduce the morbidity and mortality with untreated metabolic conditions.

In this study, hypertension was identified as the most prevalent comorbid condition among patients with SMI who were receiving psychotropic medications, further emphasizing the need for enhanced monitoring. Although there is high prevalence of metabolic syndrome in this population, routine evaluations of metabolic health are often inadequate. This gap is particularly pronounced in developing countries, where access to comprehensive healthcare services is limited [57].

In contrast our study revealed that the rate of routine health monitoring for metabolic disorders was higher compared to a literature review that reported approximately 30% of people on antipsychotics received metabolic monitoring [37]. This is an encouraging sign of progress, indicating that a greater proportion of patients engaging in routine monitoring practices, which reflects improving healthcare standards for individuals with SMI. However, these rates still leave a significant opportunity for improvement. Ensuring regular monitoring of hypertension and other metabolic parameters is crucial for early detection and management of metabolic syndrome, which can prevent the progression of severe health complications such as cardiovascular disease and diabetes. Therefore, there is a pressing need for healthcare providers to implement systematic monitoring protocols and to prioritize the health of patients with comorbid conditions to ensure they receive comprehensive care.

Routine monitoring of vital signs was conducted for a higher percentage of the patients in this study, consistent with findings from psychiatric hospitals in Ghana [46]. Vital signs, such as blood pressure, are crucial for assessing the immediate health status of patients with SMI, who are at higher risk of cardiovascular issues. However, more comprehensive metabolic monitoring including basic metabolic syndrome markers, such as glucose levels, lipid profiles, and body mass index, was inadequately performed. This gap in monitoring is concerning given the established link between psychotropic medications and metabolic disorders. Our findings align with studies conducted in South Africa and England, which also reported insufficient monitoring of these metabolic parameters [57, 58]. A previous study found that only 38% of patients on antipsychotics had their blood sugar monitored, and 23% had their cholesterol levels checked [59]. Other similar studies reported even lower rates of monitoring, with fewer than 11% of patients receiving lipid monitoring and fewer than 22% undergoing glucose assessments [60, 61]. These low monitoring rates are particularly alarming given the high prevalence of metabolic syndrome in patients with SMI. Clinical guidelines recommend that metabolic monitoring, including assessments of lipid levels and blood glucose, should be conducted at specific intervals, initially at the start of treatment, three months later, after twelve months, and then annually. These tests are essential for detecting early signs of metabolic dysfunction and enabling timely interventions. Fasting blood glucose tests, in particular, are considered one of the most reliable measures for diagnosing diabetes and monitoring glucose metabolism. In our study, however, these assessments were not routinely conducted, reflecting a significant gap in adherence to best practices for patients at high risk of metabolic syndrome. Another critical issue identified in this study was the lack of routine organ function tests. Monitoring kidney and liver function is especially important for patients taking mood stabilizers, as these medications can affect organ health over time [39].

Certain antipsychotics, such as clozapine and olanzapine, have been shown to cause the highest rates of metabolic side effects, including elevated cholesterol and triglycerides levels [62, 63]. For patients experiencing metabolic side effects, switching to antipsychotics that have a lower risk of causing such adverse outcomes is recommended [33, 34]. However, without consistent metabolic monitoring, identifying these side effects early enough to intervene remains a challenge, leaving patients vulnerable to severe complications. Furthermore, many medical records lacked documentation of metabolic monitoring, suggesting that healthcare providers may not be fully aware of the metabolic risk faced by patients with SMI [10]. This lack of awareness likely contributes to the inadequate monitoring observed in many settings, including our study.

This study also assessed the determinants of routine health monitoring practices among patients with SMI. The findings revealed that being 45 years and older, having health insurance, experiencing side effects, and the availability of laboratory testing were significantly associated with likelihood of receiving routine health monitoring. Patients whose age is 45 and older were more likely to undergo routine health monitoring compared to younger patients. This findings aligns with a similar study, which also reported that routine monitoring was more prevalent among older adults [64]. This may be due to older adults having multiple comorbidities, complications, and being on polypharmacy regimes [65], prompting health care providers to prioritize their health monitoring. The complexity of managing both physical and mental health issues in older adults’ likely leads to more frequent assessments and interventions.

Health insurance coverage was another key factor associated with increased routine health monitoring. Patients with health insurance were more likely to receive routine health assessments, likely because they faced fewer financial barriers accessing diagnostic tests. In contrast, uninsured patients may forgo necessary tests due to the cost, indicating the critical role that financial accessibility plays in determining the quality of healthcare received. Experiencing medication side effects was also a significant determinant of routine monitoring, as health care providers tend to monitor patients with side effects more closely to assess the severity and adjust treatment plans as needed. Additionally, the availability of laboratory testing at the healthcare facilities significantly impacted routine health monitoring. When laboratory tests were readily available, patients were more likely to receive appropriate assessments, whereas in settings lacking such services, patients often could not afford to seek tests from private healthcare facilities, leading to missed opportunities for monitoring.

Although further research is needed to assess the effectiveness of strategies that promote regular monitoring, patient centered programs offering more convenient and flexible options such as home based monitoring [66]. Such programs could alleviate the anxiety and discomfort patients may feel when visiting clinical settings for testing. It is also important to note that in our study setting, the healthcare system is burdened by a shortage of mental health providers, which increases the clinicians’ workload. This heavy workload may contribute to inadequate monitoring of metabolic conditions in patients with SMI, as health care providers have limited time to conduct thorough assessments [45].

### Strength and limitation

This study’s prospective follow-up design allowed for the collection of real-time data. A limitation of the study is the reliance on self-reported side effects, which may introduce recall bias and affect data accuracy. Future studies should use objective measures to reduce bias and enhance data reliability.

## Conclusion

Patients with severe mental illnesses attending the outpatient psychiatry department received inadequate routine health monitoring for metabolic disorders. Health care providers should focus on ensuring better monitoring for younger patients, those without health insurance, and those have not reported any side effects. Moreover, the government should prioritize the incorporation and development of laboratory testing facilities in hospitals. The findings from this study provide valuable insights for enhancing routine health monitoring.

## Electronic supplementary material

Below is the link to the electronic supplementary material.

Supplementary Material 1

Supplementary Material 2

## Data Availability

All the necessary data is included in the manuscript. On request, the corresponding author can give any additional details.

## Abbreviations

BMI: Body Mass Index
DMCSH: Debre Markos Comprehensive Specialized Hospital
ETB: Ethiopian Birr
SMI: Serious Mental Illness
