# Supplementary material for: Evaluation of routine health monitoring for metabolic disorders in patients with serious mental illness on psychotropic medications: a study from Ethiopia
Source: BMC Psychiatry. 2024 Nov 12;24:795. doi: 10.1186/s12888-024-06266-1 (PMC11558983; doi:10.1186/s12888-024-06266-1)
Supplement: Supplementary file 1 — Supplementary Material 1 [file 12888_2024_6266_MOESM1_ESM.docx]

**Supplementary file 1**

List of health monitoring parameters to be assessed among patients with serious mental illness taking psychotropic medications.

| Medication | Monitoring parameters | Time to monitor | | |
| --- | --- | --- | --- | --- |
|  |  | At inititation of treatment | At Titration period | At maintence period |
| Antipsychotics | Body mass index | Recommended to check | Recommendation not avialable | Weekly for the first 4-6 weeks; at least once every 4 weeks for 12 weeks,then at 6 months and annualy thereafter |
|  | Weight | Recommended to check | Recommendation not avialable | Weekly for the first 4-6 weeks; at least once every 4 weeks for 12 weeks,then at 6 months and annualy there after |
|  | Blood pressure | Recommended to check | At every followup visit | At every follow-up visit |
|  | Fasting or random glucose | Recommended to check | Recommendation not avialable | At 12 weeks, then at 6 months |
|  | Fasting or random Lipid profile | Recommended to check | Recommendation not avialable | At 12 weeks, then at 6 months |
| Atypical antipsychotics | Personal family history | Recommended to check |  |  |
|  | Weight | Recommended to check | At 2, 8, 12 weeks | In the first year: every 3 months |
|  | Body mass index | Recommended to check | At 2, 8, 12 weeks | In the First year: every 3 months |
|  | Blood pressure | Recommended to check | At 12 weeks | As clinically indicated |
|  | Fasting glucose | Recommended to check | At 12 weeks | Annually or as clinically indicated |
|  | Fasting random Lipid profile | Recommended to check | At 12 weeks | Annually or as clinically indicated |
| Lithium | Kidney function tests (Creatinine) | Recommended to check | Li level every 4-14 days | First six months: check once or twice, then every 6 months to 1 year |
|  | Thyroid function tests | Recommended to check | Recommendation not avialable | First six months: check once or twice, then every 6 months to 1 year |
|  | Liver function tests | Recommended to check | Recommendation not avialable | At 1 month, then every 3 to 6 months |
|  | Complete blood count | Recommended to check | Recommendation not avialable | At 1 month, then every 3 to 6 months |
|  | Electrolytes | Recommended to check | Recommendation not avialable | Recommendation not avialable |
| Carbamazepine | CBC; liver, kidney and thyroid function tests | Recommended to check | Not recommended | Not recommended |
|  | Complete blood count | Recommended to check | First 2 months : every 2-4 weeks. | Every 3 to 6 months |
|  | Kidney function tests | Recommended to check | Recommendation not avialable | Every 6 to 12 months |
|  | Liver function tests | Recommended to check | Recommendation not avialable | Every 6 to 12 months |
|  | Thyroid function tests | Recommended to check | Recommendation not avialable | Every 6 to 12 months |
| Valporic acid | Body mass index | Recommended to check | Recommendation not avialable | Recommendation not avialable |
|  | Blood pressure | Recommended to check | Recommendation not avialable | Recommendation not avialable |
|  | Complete blood count | Recommended to check | At one month | Every 3 to 6 months |
|  | Liver function tests | Recommended to check | At one month | Every 3 to 6 months |
|  | Electrolyte | Recommended to check | Recommendation not avialable | Recommendation not avialable |
