# Supplementary material for: Evaluation of routine health monitoring for metabolic disorders in patients with serious mental illness on psychotropic medications: a study from Ethiopia
Source: BMC Psychiatry. 2024 Nov 12;24:795. doi: 10.1186/s12888-024-06266-1 (PMC11558983; doi:10.1186/s12888-024-06266-1)
Supplement: Supplementary file 2 — Supplementary Material 2 [file 12888_2024_6266_MOESM2_ESM.docx]

**Supplementary file 2**

Data abstraction checklist for research topic: Evaluation of Routine Health Monitoring for Metabolic Disorders in Patients with Serious Mental Illness on Psychotropic Medications in Ethiopia.

**Consent to participate**

We invite you to participate in a research study focused on evaluating routine health monitoring practices among patients with serious mental illness who are on psychotropic medications. Participation is voluntary, and you may withdraw from the study at any time without any effect on your medical care or negative consequences.

The study’s objective is to evaluate current health monitoring practices and associated factors. If you agree to participate, the data collector will complete a questionnaire. This process will take approximately 5 to 10 minutes.

All information collected will remain strictly confidential and anonymous. No identifying details will be recorded or published, ensuring that your responses cannot be linked to you in any way. Your answers will be used solely for research purposes. Although there are no direct benefits to you, your input may contribute to enhancing care for other patients in the future.

For any questions or concerns about the study, please contact the researcher at +251919187613.

By signing below, you confirm your understanding of the study’s purpose and agree to participate voluntarily.

Participant’s Signature __________________________________

Date: _______________________________________________

Data collector’s name______________ Date_________________

Sign_______________

**Section 1: Socio-demographic characteristics**

1. Age __________________________________________________
2. Sex
3. Male
4. Female
5. Residence ___________________________________________________
6. **Education Level**
7. Illiterate
8. Literate with no formal education
9. Primary education
10. Secondary education
11. Higher education
12. Average monthly income in Ethiopian Birr____________
13. Do you have health insurance?
14. Yes
15. No

**Section 2: Clinical related parameters**

1. Diagnosis___________________________________________________________________
2. Is there a comorbid condition?
   1. Yes
   2. No
3. If the answer for question number 2 is ‘yes’ list the comorbid conditions ___________________________________________________________________________________________________________________________________________________________________________________________________________________________________________________________________________________________________________________________________________________________________________________________________________________________________________________________________________________________________________________________________________________________________________________________________________________________________________________________________________________________

**Section 3: Medication related parameters**

1. Health care provider specialty ________________________________________________

2. List of medications prescribed

|  | Name of medication | Dose | Frequency | Duration |
| --- | --- | --- | --- | --- |
| 1 |  |  |  |  |
| 2 |  |  |  |  |
| 3 |  |  |  |  |
| 4 |  |  |  |  |
| 5 |  |  |  |  |
| 6 |  |  |  |  |
| 7 |  |  |  |  |
| 8 |  |  |  |  |
| 9 |  |  |  |  |
| 10 |  |  |  |  |
| 11 |  |  |  |  |

1. Has the patient experienced any medication-related side effects?
   1. Yes
   2. No

**Section 4: Health monitoring practice for metabolic disorders**

1. Routine health monitoring practices assessment

|  | Medications prescribed | Health monitoring done | | List of monitoring parameters done | When monitoring was done? |
| --- | --- | --- | --- | --- | --- |
|  |  | Yes | No |  |  |
|  |  |  |  |  |  |
|  |  |  |  |  |  |
|  |  |  |  |  |  |
|  |  |  |  |  |  |
|  |  |  |  |  |  |
|  |  |  |  |  |  |
|  |  |  |  |  |  |
|  |  |  |  |  |  |
|  |  |  |  |  |  |
|  |  |  |  |  |  |
|  |  |  |  |  |  |
|  |  |  |  |  |  |
|  |  |  |  |  |  |
|  |  |  |  |  |  |
|  |  |  |  |  |  |
|  |  |  |  |  |  |
|  |  |  |  |  |  |
|  |  |  |  |  |  |
|  |  |  |  |  |  |
|  |  |  |  |  |  |
|  |  |  |  |  |  |
|  |  |  |  |  |  |
|  |  |  |  |  |  |
|  |  |  |  |  |  |

1. Avialbe laboratory tests at the hospital

______________________________________________________________________________________________________________________________________________________________________________________________________________________________________________________________________________________________________________________________________________________________________________________________________

***Thank you for your participation***
